# Chroman-4-One Derivatives Targeting Pteridine Reductase 1 and Showing Anti-Parasitic Activity

**DOI:** 10.3390/molecules22030426

**Published:** 2017-03-08

**Authors:** Flavio Di Pisa, Giacomo Landi, Lucia Dello Iacono, Cecilia Pozzi, Chiara Borsari, Stefania Ferrari, Matteo Santucci, Nuno Santarem, Anabela Cordeiro-da-Silva, Carolina B. Moraes, Laura M. Alcantara, Vanessa Fontana, Lucio H. Freitas-Junior, Sheraz Gul, Maria Kuzikov, Birte Behrens, Ina Pöhner, Rebecca C. Wade, Maria Paola Costi, Stefano Mangani

**Affiliations:** 1 Department of Biotechnology, Chemistry and Pharmacy, University of Siena, 53100 Siena, Italy; dipisa2@unisi.it (F.D.P.); landi31@unisi.it (G.L.); delloiacono3@unisi.it (L.D.I.); pozzi4@unisi.it (C.P.); 2 Department of Life Sciences, University of Modena and Reggio Emilia, Via Campi 103, 41125 Modena, Italy; chiara.borsari@unimore.it (C.B.); stefania.ferrari@unimore.it (S.F.); matteo.santucci@unimore.it (M.S.); 3 Institute for Molecular and Cell Biology, 4150-180 Porto, Portugal and Instituto de Investigação e Inovação em Saúde, Universidade do Porto and Institute for Molecular and Cell Biology, 4150-180 Porto, Portugal; santarem@ibmc.up.pt (N.S.); cordeiro@ibmc.up.pt (A.C.-d.-S.); 4 Laboratório Nacional de Biociências (LNBio), Centro Nacional de Pesquisa em Energia e Materiais (CNPEM), Campinas SP13083-100, Brazil; carolinaborsoi@gmail.com (C.B.M.); laura.alcantara@lnbio.cnpem.br (L.M.A.); fontana_vanessa@yahoo.com.br (V.F.); lucio.freitas@butantan.gov.br (L.H.F.-J.); 5 GARDE, Instituto Butantan, São Paulo SP05503-900, Brazil; 6 Fraunhofer Institute for Molecular Biology and Applied Ecology Screening Port, D-22525 Hamburg, Germany; Sheraz.Gul@ime.fraunhofer.de (S.G.); Maria.Kuzikov@ime.fraunhofer.de (M.K.); Birte.Behrens@ime.fraunhofer.de (B.B.); 7 Molecular and Cellular Modeling Group, Heidelberg Institute for Theoretical Studies, 69118 Heidelberg, Germany; ina.poehner@h-its.org (I.P.); rebecca.wade@h-its.org (R.C.W.); 8 Center for Molecular Biology (ZMBH), DKFZ-ZMBH Alliance, Heidelberg University, 69120 Heidelberg, Germany; 9 Interdisciplinary Center for Scientific Computing (IWR), Heidelberg University, 69120 Heidelberg, Germany; 10 Magnetic Resonance Center CERM, University of Florence, 50019 Sesto Fiorentino (FI), Italy

**Keywords:** pteridine reductase 1, *Trypanosoma brucei*, *Leishmania* spp., chroman-4-one, chromen-4-one, crystallographic studies

## Abstract

Flavonoids have previously been identified as antiparasitic agents and pteridine reductase 1 (PTR1) inhibitors. Herein, we focus our attention on the chroman-4-one scaffold. Three chroman-4-one analogues (**1**–**3**) of previously published chromen-4-one derivatives were synthesized and biologically evaluated against parasitic enzymes (*Trypanosoma brucei* PTR1–*Tb*PTR1 and *Leishmania major–Lm*PTR1) and parasites (*Trypanosoma brucei* and *Leishmania infantum*). A crystal structure of *Tb*PTR1 in complex with compound **1** and the first crystal structures of *Lm*PTR1-flavanone complexes (compounds **1** and **3**) were solved. The inhibitory activity of the chroman-4-one and chromen-4-one derivatives was explained by comparison of observed and predicted binding modes of the compounds. Compound **1** showed activity both against the targeted enzymes and the parasites with a selectivity index greater than 7 and a low toxicity. Our results provide a basis for further scaffold optimization and structure-based drug design aimed at the identification of potent anti-trypanosomatidic compounds targeting multiple PTR1 variants.

## 1. Introduction

In modern drug discovery, crystallography is an essential tool for rapid scaffold optimization through structure-based drug design. It provides a basis for the application of computational methods to design ligands for the targeted proteins [1]. Thus, the determination of novel crystal complexes of parasitic proteins and ligands represents a starting point for a medicinal chemistry approach to the discovery of novel antiparasitic compounds. Trypanosomatid parasites are the etiologic agents of serious human and animal vector-borne infections, such as Human African Trypanosomiasis (HAT, also known as sleeping sickness) and Leishmaniasis. The bloodstream form of the protozoan parasite *Trypanosoma brucei* (*T. brucei*) causes HAT [2], while *Leishmania* spp. infects macrophages and causes different clinical forms ranging from cutaneous lesions to potentially fatal visceral infections [3]. Since vaccines to prevent HAT and Leishmaniasis are currently not available, the control of these diseases is essentially based on chemotherapy. Almost all the drugs used to combat parasitic infections were discovered decades ago and nowadays drug resistance is a major threat. Moreover, the drugs currently available present several problems such as high toxicity, limited efficacy, parenteral administration regimens and long periods of treatment [4,5]. Thus, the discovery of novel, safe and effective drugs is an unmet medical need and an ongoing challenge. Drug discovery for neglected tropical diseases relies both on phenotypic screening and target-based approaches [6,7]. Dihydrofolate reductase (DHFR) is a well-established target for the treatment of bacterial infections and some parasitic diseases, such as malaria [8]. The classical inhibitors of DHFR have reduced activity against *Leishmania* and *Trypanosoma* due to the upregulation of a gene encoding the dihydronicotinamide adenine dinucleotide phosphate (NADPH)-dependent pteridine reductase 1 (PTR1). PTR1 is present in *Leishmania* spp. and *Trypanosoma brucei* parasites, but not in the human cells. It is able to reduce both unconjugated and conjugated pterins and provides a metabolic bypass to alleviate DHFR inhibition [9,10,11,12,13,14]. PTR1 is considered a promising target for the development of novel antitrypanosomal and antileishmanial candidates and has recently been genetically validated as a drug target in *T. brucei* [15]. In the literature, different scaffolds, such as pteridine [16], pyrrolopyrimidine [17,18] and benzimidazole [19,20], have been reported to bind in the biopterin binding site and to inhibit PTR1 activity. In our previous work, we have shown that chromen/chroman-4-ones were promising scaffolds for the development of PTR1 inhibitors and antiparasitic agents. Four crystal structures of *Trypanosoma brucei* pteridine reductase 1 (*Tb*PTR1) in complex with three flavonols and one flavanone had been solved, providing essential tools for the development of improved inhibitors [14]. Our previous attempts to obtain X-ray crystal structures of *Leishmania major* pteridine reductase 1 (*Lm*PTR1) in complex with inhibitors failed and no crystal structure complexes of *Lm*PTR1-flavonoids have been reported in the literature to date.

**Figure 1:**
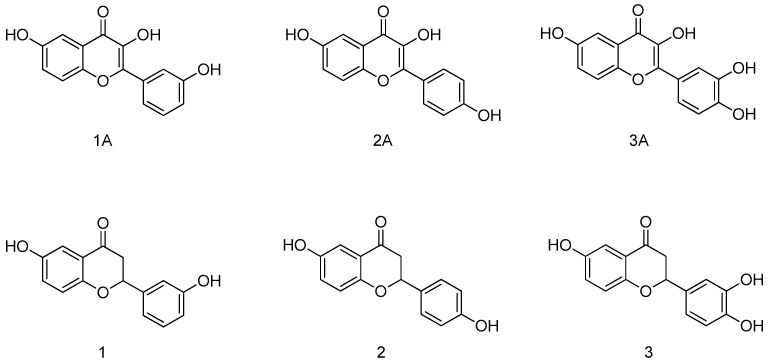
Chemical structures of the previously published flavonols [14] (**1A**–**3A**) and of the flavanone analogues studied in the present work (**1**–**3**).

In the present work, we synthesized three flavanones (**1**–**3**) that are analogues to our previously published flavonols (**1A**–**3A**) [14] (Figure 1) in order to compare their binding mode in the PTR1 active site and their biological activity. The crystal structure of *Tb*PTR1 in a ternary complex with compound **1** was solved and the first crystal structures of *Lm*PTR1-flavanone (compounds **1** and **3**) ternary complexes were obtained. Computational studies were also performed to explain the differences between the binding modes of the chromen-4-one and the chroman-4-one moiety. We evaluated the compounds for inhibitory activity against PTR1 and for antiparasitic activity against the parasites *T. brucei* and *Leishmania infantum* (*L. infantum*). Finally, a wide panel of in vitro toxicological studies to assess the safety of the proposed scaffold were undertaken.

## 2. Results and Discussion

### 2.1. Synthesis and Inhibitory Activity towards PTR1

**Scheme 1:**
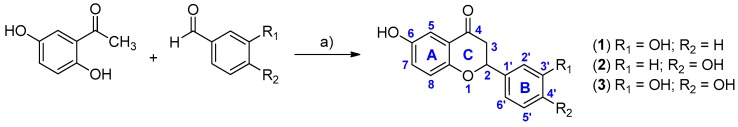
Synthesis of compounds **1**–**3**. *Reaction conditions*: (a) SOCl_2_, EtOH, r.t.

Previously [14], we had synthesized a library of 16 flavonols and compound **1A** turned out to be the most potent *Tb*PTR1 inhibitor. In the present work, we designed and synthesized compounds **1**–**3**, analogues of **1A**–**3A** respectively, in order to compare the binding modes and the affinities of flavanones with those of flavonols. Compounds **1**–**3** were synthesized by condensation between substituted acetophenone and benzaldehydes in the presence of thionyl chloride in ethanol, as previously reported (Scheme 1) [21]. The compounds were characterized by ^1^H-NMR, ^13^C-NMR and mass analysis and the data are reported in the Supporting Information (p. S2).

The three flavanones were investigated for their activity against *Tb*PTR1 and *Lm*PTR1. The data were compared with those of compounds **1A**–**3A**. The results are reported in Table 1.

Among the three synthesized flavanones, compound **1** was the most potent derivative against *Tb*PTR1, with a percentage of inhibition of 81% and an IC_50_ value of 31 µM. All three flavanones show a percentage of inhibition of *Lm*PTR1 greater than 50%, with compounds **2** and **3** being the most active (IC_50_ 35 and 36 µM, respectively).

### 2.2. Crystallographic Structures of LmPTR1 and TbPTR1 in Complex with Flavanones

The compounds were subjected to crystallization trials to gain further information for structure-based drug design. Ternary complex structures of *Lm*PTR1-NADP^+^ with compounds **1** and **3**, and *Tb*PTR1-NADP^+^ with compound **1** were obtained at resolutions ranging from 1.70 Å to 2.35 Å. Data collection and processing statistics are reported in Tables S2 and S3 of the Supporting Information.

**Figure 2:**
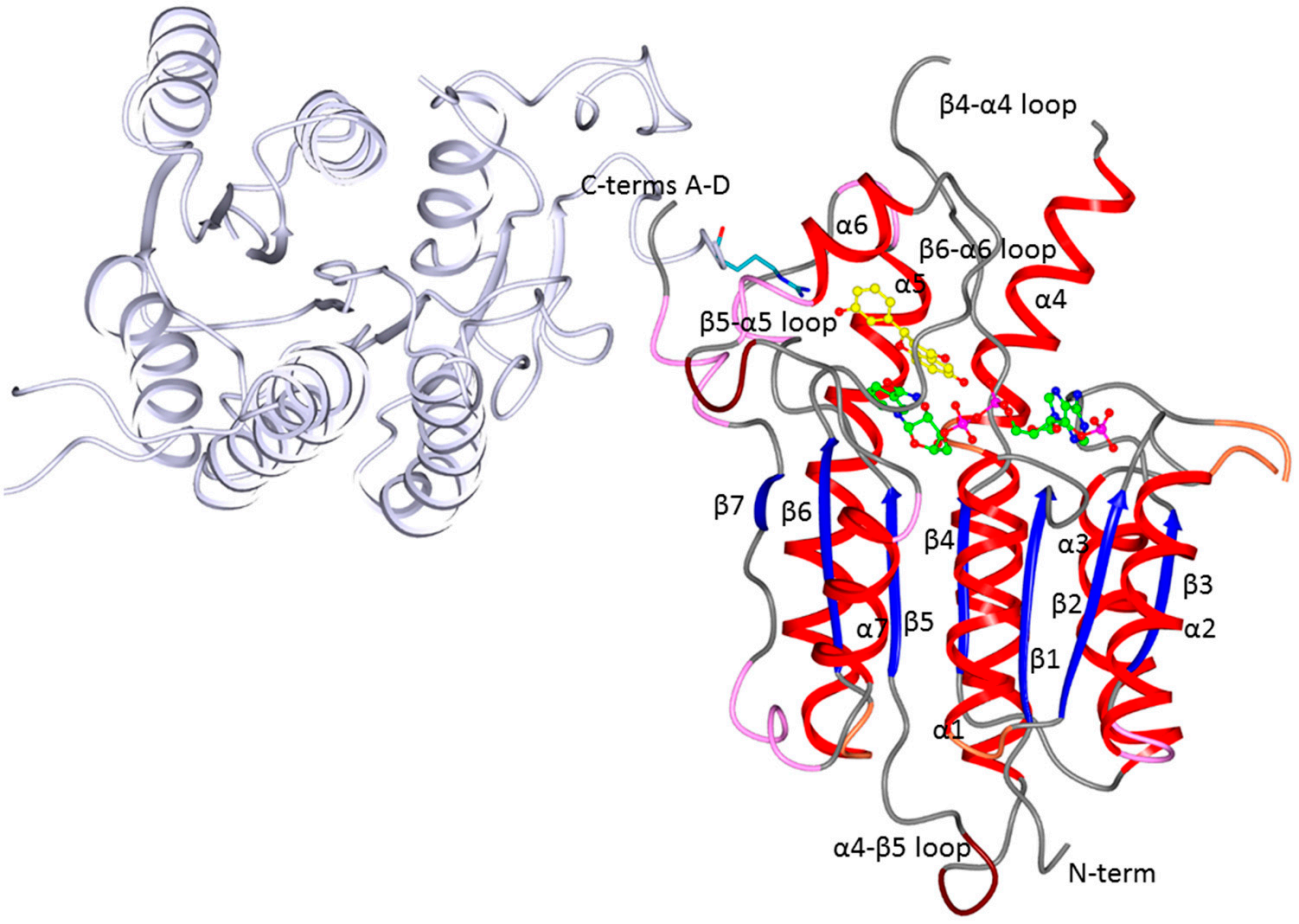
Ribbon diagram of the *Lm*PTR1 subunit A showing the tertiary and secondary structures as well as the location of the cofactor (green ball and sticks) and an inhibitor (Compound **1**; yellow ball and stick) in the active site. The subunit D (pale lilac cartoon) contributing to the active site of the A subunit with Arg287 (cyan sticks) is also shown. The helices of subunit A are colored red, the β-strands blue, and the secondary structure elements are numbered following the sequence. *Tb*PTR1 has the same fold and topology as *Lm*PTR1.

PTR1 is a functional homotetramer with four catalytic sites. Each subunit has a single α/β structured domain with the typical topology of short-chain dehydrogenases/reductases (SDR) and is characterized by a seven-stranded parallel β-sheet sandwiched by three α-helices on each side, that represents the classical dinucleotide-binding motif known as the Rossmann fold. The active site (about 30 Å × 22 Å × 15 Å) is mainly formed by a single chain, and is blocked at one end by the C-terminal residues of an adjacent subunit. The catalytic centre is formed by residues from the C-termini of β4 and α5, by the two loops connecting β5-α5 and β6-α6, and the nicotinamide of the cofactor, which creates the floor of the active site [10,22] (Figure 2). The highly conserved Ser-Tyr-Lys catalytic triad, typical of the SDR family, is replaced by Asp-Tyr-Lys in both *Tb*PTR1 and *Lm*PTR1 (Asp181, Tyr194 and Lys198 in *Lm*PTR1; Asp161, Tyr174 and Lys178 in *Tb*PTR1). The biopterin binding pocket is further completed by the aromatic side chain of Phe113 in *Lm*PTR1 and Phe97 in *Tb*PTR1, forming an overhang under which the substrate binds through a characteristic π-sandwich interaction involving the nicotinamide moiety of the cofactor [23].

The asymmetric units of all three structures presented here contain one PTR1 homotetramer with identical subunits within experimental error (pairwise root mean square deviation, rmsd, values, calculated on Cα atoms, ranging from 0.26 Å to 0.38 Å for *Lm*PTR1-NADP^+^-compound **1**, 0.21 Å to 0.27 Å for *Lm*PTR1-NADP^+^-compound **3** and 0.14 to 0.34 Å for *Tb*PTR1-NADP^+^-compound **1**).

Structural alignments of the Cα atoms of each ternary complex of *Lm*PTR1 and *Tb*PTR1 and the respective PTR1-NADP^+^ binary complexes (Protein Data Bank (PDB) code 2BFO for *Lm*PTR1 and unreleased structures from our laboratory for *Tb*PTR1) resulted in rmsd values ranging from 0.18 Å to 0.39 Å, indicating that inhibitor binding does not cause large conformational changes of the enzyme. Even the superposition of the *Lm*PTR1 and *Tb*PTR1 ternary complexes with the ternary structures present in the PDB reveals no major structural changes. The high degree of structural similarity also extends to many side chains, the conformation of the cofactor and the positions of several water molecules. This is a clear indicator of the high level of structural conservation of the protein, irrespective of the binding of substrate, product or inhibitors.

**Figure 3:**
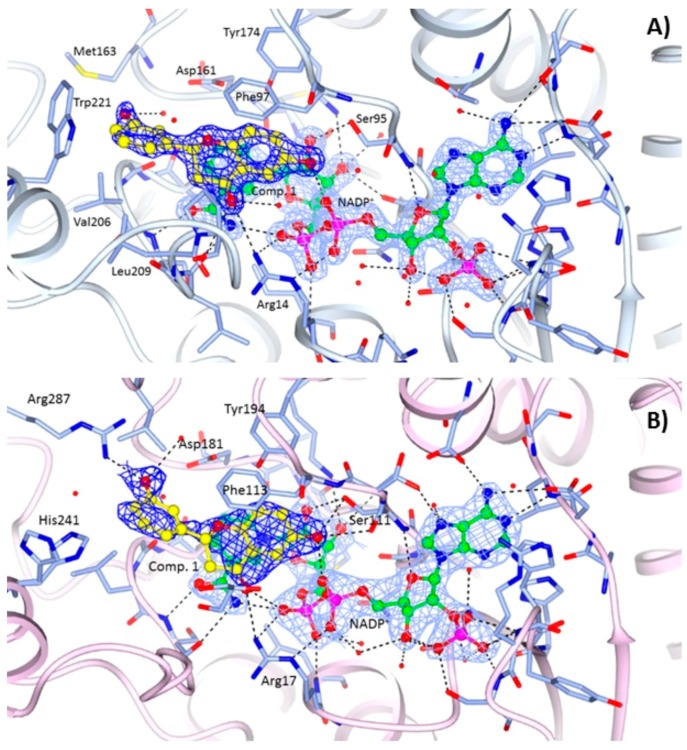

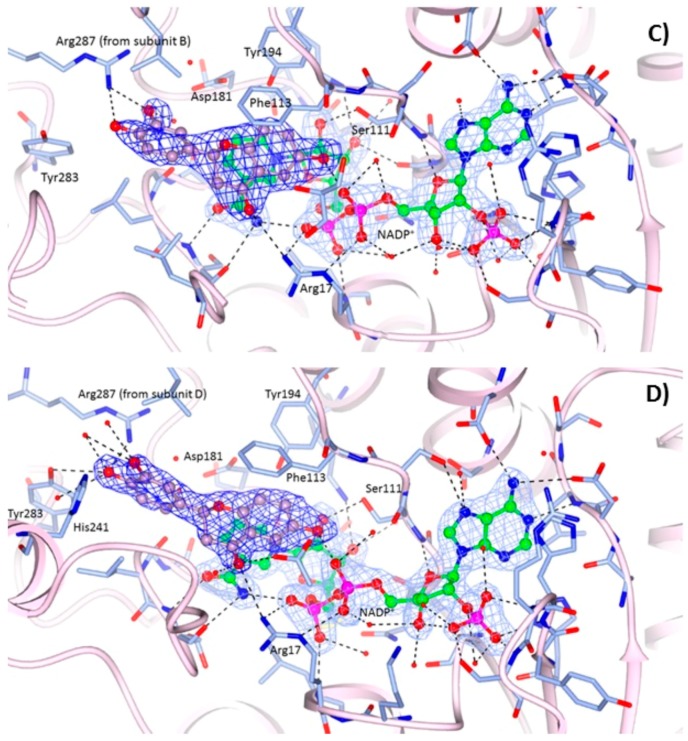
Binding of the NADP^+^ cofactor (ball and sticks, green carbon atoms) and compounds **1** (ball and sticks, yellow carbon atoms) in the active site of: (**A**) *Tb*PTR1 (grey ribbons); and (**B**) *Lm*PTR1 (pink ribbons). Binding of the NADP^+^ cofactor (ball and sticks, green carbon atoms) and compound **3** (ball and sticks, purple carbon atoms) in the active site of: (**C**) *Lm*PTR1 subunit C, and (**D**) *Lm*PTR1 subunit A. The 2Fo−Fc electron density maps corresponding to the inhibitors (dark-blue wire) and NADP^+^ (light-blue wire), contoured at the 1σ level, are shown. H-bonds involving the cofactor and inhibitor are shown as dashed lines. It is worth noting the different orientation and environment of the compound **3** catechol ring between subunits C and A of *Lm*PTR1.

***Tb*PTR1-NADP^+^-1 complex**. The structure was determined at 1.70 Å resolution. Ligand placement and key interactions within the active site cavity are reported in Figure 3A. The chroman-4-one moiety of compound **1** is involved in a π-sandwich between the nicotinamide of NADP^+^ and Phe97, and the ether O1 points towards the side chains of Asp161 and Tyr174. The hydroxyl group in position 6 establishes a H-bond with the oxygen atom O2A of NADP^+^ β-phosphate and a weaker one (3.45 Å) with the Ser95 side chain. The carbonyl group in position 4 is H-bonded to the amino group of Arg14 and linked to the oxygen atom (O2A) of the cofactor through a water molecule. The phenol linked to position 2 of the chroman-4-one is located in a prevalently hydrophobic pocket lined by Met163, Val206, Leu209, Met213, Trp221 and Leu263, and is able to make a T-shaped stacking interaction with the side chain of Trp221 and a water-mediated interaction with the side chain of Asp161. Even though the soaking procedure was performed using a racemic mixture only the R-enantiomer has been observed in the active site cavity (no evidences for the S-enantiomer in the electron density map as shown in Figure 3A).

***Lm*PTR1-NADP^+^-1** and ***Lm*PTR1-NADP^+^-3 complexes.** The structures have been determined at 2.35 Å and 2.10 Å resolution, respectively. The orientations of compounds **1** (Figure 3B) and **3** (Figure 3C,D) in the catalytic pocket of *Lm*PTR1 resemble that of compound **1** in the *Tb*PTR1 active site (Figure 3A). Moreover, as reported above for the complex with *Tb*PTR1, only the R-enantiomer of the compounds has been observed within the catalytic cavity of *Lm*PTR1. The chroman-4-one scaffold establishes a π-sandwich between the nicotinamide of NADP^+^ and Phe113 and most of the interactions with the surrounding residues are retained across the two species: the hydroxyl group in position 6 of both compounds is located within H-bonding distance of the β-phosphate oxygen atom O2A of NADP^+^, and Ser111 side chain. Compound **1** is also able to establish a polar contact with the Ser111 side chain (Figure 3B). In both compounds, the carbonyl group in position 4 is H-bonded to the NH_2_ group of Arg17. As in *Tb*PTR1, the phenol moieties are located in a prevalently hydrophobic pocket lined by Leu188, Leu226, Leu229, His241 and Arg287 (for clarity, leucine residues are not labelled in Figure 3B–D). The phenol ring of compound **1** is able to establish a strong H-bond with the Arg287 side chain, which points into the catalytic pocket from an adjacent subunit of the tetramer, and a water-mediated interaction with Asp181. In the complex *Lm*PTR1-**3**, the involvement of the *m*-hydroxyl group of the catechol ring in the interaction with the NH_2_ group of Arg287 is visible only in chains C and D (Figure 3C), whereas in chains A and B (Figure 3D), due to a flip of the catechol ring of about 120°, the *p*-hydroxyl group interacts with the side chain of Tyr283.

Irrespective of the compound in the catalytic pocket, different conformations of His241 in *Lm*PTR1, at the corresponding position of Trp221 of *Tb*PTR1, were observed in the tetramer, indicating a high flexibility of this residue which delimits the catalytic pocket.

### 2.3. Docking Studies and Crystal Structure Comparison

The binding modes of the flavanones with those of their corresponding flavonols in *Tb*PTR1 and *Lm*PTR1 were compared by performing docking studies with a ligand-based constraint in order to favor binding modes closely related to the crystallographic reference modes as previously described [14].

**Figure 4:**
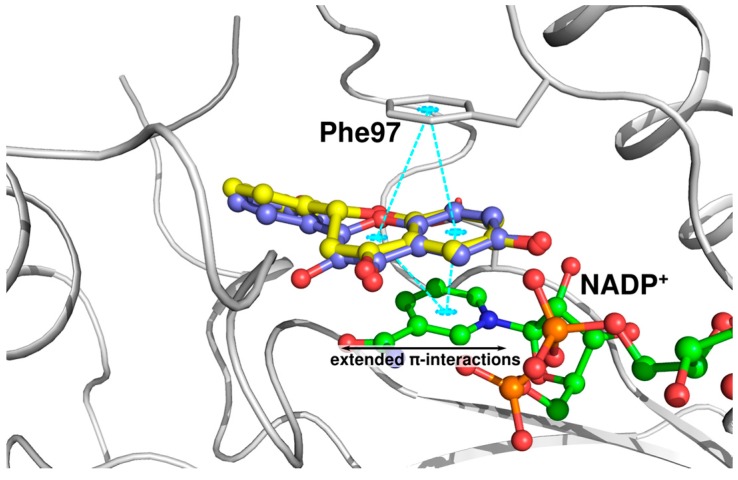
Comparison of the binding modes of compound **1** (ball-and-stick, yellow carbons) and compound **1A** (lilac carbons) in *Tb*PTR1 (gray cartoon). The NADP^+^ cofactor (in ball-and-stick, green carbon atoms) and Phe97 are included for reference. Possible π-stacking interactions are indicated by cyan dotted lines.

The crystal structures of *Tb*PTR1 and *Lm*PTR1 show almost identical binding modes for compound **1** (Figure 3A,B). Interestingly, compound **1** is about twice as potent against *Tb*PTR1 as against *Lm*PTR1 (IC_50_ *Tb*PTR1 = 31 µM, *Lm*PTR1 = 57 µM). The *Lm*PTR1 binding pocket is smaller than the *Tb*PTR1 pocket, since one residue from the neighboring subunit, which is pointing into the active site, namely Arg287 of *Lm*PTR1, is replaced by the smaller His267 in *Tb*PTR1. In *Lm*PTR1, ring B is in close proximity to Arg287 and adopts a slightly different orientation allowing a hydrogen bond between 3′-OH and Arg287 to be formed. Notably, in *Tb*PTR1, no direct interaction with His267 is possible and the 3′-OH can only interact with a fully conserved water molecule, and ring B is stabilized by a T-shaped stacking interaction with Trp221. The corresponding His241 of *Lm*PTR1 does not allow a similar stabilizing interaction. We conclude that the gating role of Trp221 in *Tb*PTR1, leading to a more closed pocket, is favorable for activity. The *Lm*PTR1 pocket remains more open and more accessible for bulk water; the placement of a hydrophobic ligand moiety in a solvent-exposed environment could affect the binding of compound **1** to *Lm*PTR1, leading to the lower affinity. The binding modes of compound **1** and flavonol **1A** are almost identical. Nevertheless, compound **1A** is about eight-fold more active against *Tb*PTR1 than compound **1** (IC_50_ *Tb*PTR1 4.3 and 31.0 µM, respectively). As shown in Figure 4, both the chromanone of compound **1** and the chromenone of compound **1A** occupy the stacking position between Phe97 and the cofactor nicotinamide. Compound **1A** can form extended aromatic interactions with the π-system of the cofactor; on the contrary, only the aromatic ring A of compound **1** can interact in a similar manner, while ring C contributes solely to hydrophobic stabilization. Moreover, ring C is located in proximity of the rather polar carboxyamide group of NADP^+^, which may further explain why the binding of a chromanone (compound **1**) is less favorable than that of a chromenone (compound **1A**). This difference in activity is also observed in *Lm*PTR1 (IC_50_ *Lm*PTR1 compound **1A**: 12.5 µM and compound **1**: 57.0 µM).

Compound **3A** is less active than **1A** (IC_50_ *Tb*PTR1 = 38.0 and 4.3 µM and IC_50_ *Lm*PTR1 = 35.0 and 12.5 µM, respectively). According to the crystal structures, compounds **1A** and **3A** show identical binding modes in *Tb*PTR1; however, the 4′-OH is solvent-exposed and it does not establish hydrogen bonds. The same pattern is observed for the inhibitory activity of the corresponding flavanones towards *Tb*PTR1 (compounds **1** and **3**, IC_50_ *Tb*PTR1 = 31 µM and 82 µM) leading to the suggestion that the flavanone compounds may share a similar binding mode. This idea is supported by our docking studies, indicating only minor reorientation of the non-interacting 4′-OH in some docking solutions (data not shown). However, due to the loss of aromatic stacking capability with the chroman-4-one ring (Figure 4), compound **3A** is again more active than compound **3** (IC_50_ *Tb*PTR1 = 38 µM and 82 µM, respectively). On the other hand, the crystal structure of *Lm*PTR1 with compound **3** reveals a binding mode different from that of compound **1**. In the latter, ring B is straightened up and hydrogen bonds are established either with Arg287 from the neighboring subunit or with Tyr283 and water. The difference in activity towards *Lm*PTR1 between flavonol **3A** and flavanone **3** is negligible (IC_50_ *Lm*PTR1 = 35 µM and 36 µM,) and only subtle differences in the binding modes are observed in the two different receptors. For example, His241 lies at about 4 Å from ring B of **3** (see Figure 3D) and is part of an alpha-helix adjacent to the substrate loop. Thus, different substituents in the *meta* position of ring B may promote additional interactions of this helix with the ligand and stabilize the substrate loop in a more closed conformation, prohibiting the solvent exposure of the chroman-4-one/chromen-4-one.

**Figure 5:**
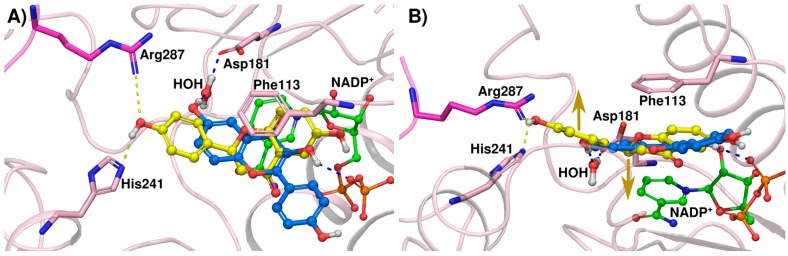
(**A**) Docking poses of compound **2** (ball-and-stick, yellow carbons) and **2A** (blue carbons) in *Lm*PTR1 (pink cartoon with Arg287 from the neighboring subunit shown in magenta sticks; NADP^+^ cofactor in ball-and-stick with green carbons). Interactions are indicated as dashed lines colored according to the interacting compounds’ carbon color. Interacting residues are shown in stick representation; (**B**) As (**A**) but rotated by approx. 90°. The yellow arrows indicate the kink in the ligand that is introduced by the chromanone ring system.

The rather elongated, largely planar structure of compound **2A** did not fit in the *Lm*PTR1 active site with a classical binding mode. The difference in activity between the inactive compound **2A** and the corresponding flavanone **2** (IC_50_ *Lm*PTR1 compound **2** = 35 µM; compound **2A**: no inhibition at 50 µM) may be explained by a different mode of binding of the kinked chroman-4-one as shown in Figure 5. In fact, compound **2** adopts the typical binding mode in the *Lm*PTR1 pocket with the 4′-OH in hydrogen bonding distance to His241 and Arg287, while compound **2A** contacts the fully conserved water molecule bridging to Asp181 with the 6-OH of the chromen-4-one. As a consequence, ring B of compound **2A** is located close to the cofactor and rather solvent-exposed. Also in *Tb*PTR1, flavanone **2** shows slight activity whereas compound **2A** is completely inactive (IC_50_ *Tb*PTR1 compound **2** = 133 µM; compound **2A**: no inhibition at 50 µM). The 4' OH does not have suitable hydrogen bonding partners to allow major stabilization of the binding mode in *Tb*PTR1, thus even the kinked conformation of the chroman-4-one core is not able to introduce new direct contacts, which is in agreement with the overall low activity.

### 2.4. Biological Profile of Compounds ***1**–**3***

In order to evaluate whether the chroman-4-one is a safe scaffold for a drug discovery program, the in vitro toxicological properties for the three compounds were determined. The inhibitory activity towards the hERG potassium channel, Aurora B kinase and five cytochrome P450s (CYP1A2, CYP2C9, CYP2C19, CYP2D6 and CYP3A4) were assessed as well as the mitochondrial toxicity and cytotoxicity (measured as A549/W1-38 cell growth). The compounds showed a safe profile: they are neither mitotoxic nor cytotoxic and they do not inhibit hERG and Aurora B kinase. Only compound **1** inhibited a single cytochrome P450 isoform (CYP2C19) at more than 70%. The data are reported in Table S4 of the Supporting Information.

Compounds **1**–**3** were tested against the bloodstream form of *T. brucei* at 10 µM and against *L. infantum* amastigotes at 50 µM, since it is usually more difficult to find antileishmanial hits. Dose response curve studies for compounds **1**–**3** against *T. brucei* were performed. Compounds **1** and **2** were the most active molecules against *T. brucei* with EC_50_ values of 12.6 ± 1.7 and 13.0 ± 1.8 µM. Compound **3** showed an EC_50_ value against *T. brucei* of 34.8 ± 1.1 µM. The compounds were assessed for cytotoxicity on THP-1 macrophage-like cells to determine the NOAEL (no observed adverse effect level). All the compounds presented a NOAEL higher than 100 µM. The data are shown in Table S5 of the Supporting Information. The selectivity index (SI), given by the ratio between the CC_50_ toward THP-1 and the EC_50_ toward *T. brucei*, was higher than 7 for compounds **1** and **2**. Compounds **1** and **2** also weakly inhibited *L. infantum* cell growth (% inhibition at 50 µM: 31% and 29%, respectively), while compound **3** was inactive against *L. infantum*.

Among the chroman-4-one inhibitors, compound **1** was the most potent derivative against both *T. brucei* and *L. infantum*. The data for compound **3** support the involvement of PTR1 inhibition in the parasitic activity. At the enzyme level, there is a 2.5-fold activity difference between compounds **1** and **3**, with compound **1** being the most potent inhibitor of *Tb*PTR1. At the biological level, the difference in potency between compounds **1** and **3** is 2.8-fold, suggesting that PTR1 might be one of the molecular targets. Compound **2** does not follow this apparent trend since it is not a good inhibitor of *Tb*PTR1, but it shows antiparasitic activity comparable to that of compound **1**. Therefore, its potency against the *T. brucei* parasite might result from effects on other protein targets.

## 3. Materials and Methods

### 3.1. General Information

All commercial chemicals and solvents were reagent grade and were used without further purification. Reaction progress was monitored by thin layer chromatography (TLC) on pre-coated silica gel 60 F254 plates (Merck KGaA, Darmstadt, Germany) and visualization was accomplished with UV light (254 nm). ^1^H- and ^13^C-NMR spectra were recorded on a Bruker FT-NMR AVANCE 400 (CIGS, Centro Interdipartimentale Gradi Strumenti, Modena, Italy). Chemical shifts are reported as δ values (ppm) referenced to residual solvent (CHCl_3_ at δ 7.26 ppm, dimethyl sulfoxide (DMSO) at δ 2.50 ppm, MeOD at δ 3.31 ppm); *J* values were given in Hz. When peak multiplicities are given, the following abbreviations are used: s, singlet; d, doublet; t, triplet; q, quartet; m, multiplet; br, broadened signal. Silica gel Merck (60–230 mesh) was used for column chromatography. The reaction progress was monitored by TLC (Merck F-254 silica gel). Mass spectra were obtained on a 6520 Accurate-Mass Q-TOF LC/MS and 6310A Ion Trap LC-MS(n) (CIGS, Centro Interdipartimentale Gradi Strumenti, Modena, Italy). The detailed nuclear magnetic resonance (NMR) and mass data of the synthesized compounds are reported in the Supporting Information (p. S2).

### 3.2. General Procedure for the Synthesis of Compounds ***1**–**3***

To a stirred mixture of 2′,5′-dihydroxyacetophenone (0.30 g, 1.97 mmol) and the appropriate aldehydes (1 eq.) in absolute ethanol (2 mL), thionyl chloride (120 µL) was added dropwise over 5 min. The reaction was stirred at room temperature for 6 h. Ethanol and excess of thionyl chloride were removed under reduced pressure on a rotary evaporator. Column chromatography was carried out to purify the desired product (eluent system: cyclohexane/ethyl acetate 9.8/0.2).

### 3.3. Computational Methodology

Compounds were generated with the LigPrep routine of Schrödinger Maestro [24] as previously described [14]. Important structural water sites in the crystal structures were identified with a WatCH clustering approach [25] for both *Lm*PTR1 and *Tb*PTR1 as previously published [14]. Docking grid preparation and docking studies followed the previously published methodology for docking with Glide [14,26,27,28]. Two crystal structures are reported in the present work, namely PDB code 5K6A for docking to *Tb*PTR1 and 5L4N for *Lm*PTR1.

The crystal structures of *Tb*PTR1 have oxo-cysteine at residue 168. To assess the possible impact of the oxo-modification on the ligand binding pose, the complex of *Tb*PTR1 was prepared with compound **1** twice: once including the modification and once with a mutation to a normal cysteine using the program PYMOL [29]. However, we found no major impact of this modification on our docking results.

### 3.4. Protein Purification and Kinetic Profile Characterization

*Tb*PTR1 and *Lm*PTR1 were purified as reported in literature [14]. The enzyme kinetic profile of each selected protein was characterized by a spectrophotometric assay. The experimental assay consists of enzyme kinetic reaction monitoring at 340 nm for a total time of 180 s. The enzyme kinetic activity was measured at *steady-state* concentration of 7,8-dihydro-l-biopterin (H2B) and NADPH substrates equal to 50 and 120 µM and varying the enzyme concentration in the 0.001–0.006 µM and 0.005–0.010–0.015–0.020–0.025–0.030 µM range for *Lm*- and *Tb*PTR1, respectively. The resulting *k*_cat_ values were equal to 0.68 (s^−1^) and 7.2 (s^−1^) for *Tb*- and *Lm*PTR1, respectively. For both *Tb*PTR1 and *Lm*PTR1, the *K*_m_ value evaluation assay for H2B- and NADPH-substrates was performed in the 0.3-0.6–0.9–1.2–2.4–4.8 µM and 3.0–6.0–12.0–40.0–90.0–190.0 µM concentrations range, respectively. The obtained H2B- and NADPH-*K*_m_ data were equal to 5.5 µM, 6.8 µM and to 10.5 µM, 16.0 µM for *Lm*- and *Tb*PTR1 respectively, and thus consistent with the data previously published [14].

### 3.5. TbPTR1 and LmPTR1 Enzyme Assay

Compounds **1A**–**3A** were evaluated as reported in our previous paper [14]. The novel synthesized compounds **1**–**3** were tested towards *Tb*PTR1 and *Lm*PTR1 using the in vitro *Tb*PTR1/*Lm*PTR1 enzyme reporter assay. The experimental procedure consists of monitoring the non-enzymatic reduction reaction of cytochrome-c at 550 nm, related to the reduction reaction of H2B to tetrahydrobiopterin (H4B) by using NADPH as the cofactor. *Tb*PTR1 and *Lm*PTR1 enzyme activity was assayed in a buffer containing 20 mM sodium citrate (pH 6.0) in a final experimental volume equal to 600 µL. The final reaction mixture contained different substrates added in the following order: *Tb*PTR1/*Lm*PTR1 (0.018 µM/0.002 µM) in 20 mM Tris-HCl, glycerol 10% (*v*/*v*), pH 7.5; H2B at a final concentration equal to 0.3 µM; cytochrome-c (80 µM); Milli-Q water to a final volume of 600 µL and NADPH to a final concentration of 500 µM. Before the addition of NADPH, the reaction mixture was incubated for 10 min at 30 °C and 80 RPM. To allow the starting of the reaction, NADPH was added and the enzyme reaction was detected for a total time of 10 min at 30 °C, 550 nm. The inhibition profile characterization of compounds **1**–**3** was performed with a DU-640 spectrophotometer (Beckman Coulter, Brea, CA, USA). Each compound was assayed in duplicate against *Tb*/*Lm*PTR1 target enzymes in the 0–10–25–50–80–150 µM concentrations range. At the different concentration values, the compounds were added to the reaction mixture (enzyme, H2B, cytochrome-c in 20 mM sodium citrate buffer and Milli-Q water) and incubated for 10 min at 30 °C, 80 RPM. The reaction was initiated by the addition of NADPH (500 µM) and monitored for 10 min at 30 °C, 550 nm. The absorbance values were processed by software analysis and transformed into the corresponding slopes. The percentages of inhibition and the IC_50_ values were determined.

### 3.6. Crystallization

*Tb*PTR1. Crystals of *Tb*PTR1 were obtained by the vapor diffusion sitting drop technique at room temperature [30]. Drops were prepared by mixing equal volumes of protein (*Tb*PTR1 6–10 mg·mL^−1^ in 20 mM Tris-HCl pH 7.5 and 10 mM DTT) and precipitant (1.5–2.5 M sodium acetate and 0.1 M sodium citrate pH 5.0) solutions and equilibrated over 600 µL of reservoir. Well-ordered monoclinic crystals grew in several days.

The ternary *Tb*PTR1-cofactor-compound **1** complex was prepared by diffusion soaking of a 2 mM solution of the inhibitor (dissolved in a 1:1 mixture of 1,4-dioxane and water) into pre-formed crystals of the native enzyme for 4 h. Crystals were then transferred in a cryo-protectant, prepared by adding 30% glycerol to the precipitant solution, and flash frozen in liquid nitrogen.

*Lm*PTR1. Crystals of the binary complexes of *Lm*PTR1 and NADPH were obtained as previously described [23], with minor modifications. The protein (12.5 mg·mL^−1^) was 10-fold diluted in 20 mM sodium acetate pH 5.3 containing 1 mM NADPH and 20 mM DTT, incubated at 4 °C for 1.5 h and later concentrated up to the starting volume with centrifugal concentrators (cutoff 10 kDa). The resulting sample was used to obtain crystals with the hanging-drop technique [30]. A drop containing 2 µL complex solution plus 2 µL of a reservoir solution consisting of 12% PEG 4600, 100 mM sodium acetate buffer pH 5.5 and 120–160 mM calcium acetate was set up against reservoir solution at 293 K. The crystals grew as clumps of thin fragile rods.

The inhibitor-bound complexes of *Lm*PTR1 with compounds **1** and **3** were prepared by diffusion soaking of a 2 mM solution of the inhibitors (dissolved in a 1:1 mixture of 1,4-dioxane and water) into pre-formed crystals of the native enzyme for 4.5 h.

Fragments of the crystal rods were cleaved for analysis, cryopreserved by transferring into a solution consisting of 70% reservoir solution and 30% glycerol and flash-cooled in liquid nitrogen.

### 3.7. Data Collection, Structure Solution and Refinement

X-ray diffraction data of *Tb*PTR1 ternary complex with NADP^+^ and compound **1** were collected at Elettra Synchrotron (Trieste, Italy) on beamline XRD-1, equipped with a Pilatus 2 M detector, using a wavelength of 1.0000 Å, a Δϕ of 1° and an exposure time of 15 s per image. Diffraction data of the ternary complex of *Lm*PTR1-NADP^+^-**1** were collected at Diamond Light Source (DLS, Didcot, UK) on beamline I04, equipped with a Pilatus 6M-F detector, using a wavelength of 0.9795 Å, a Δϕ of 0.2° and an exposure time of 0.2 s per image. Diffraction data of the complex *Lm*PTR1-NADP^+^-**3** were collected at Elettra Synchrotron (Trieste, Italy) on beamline XRD-1, equipped with a Pilatus 2M detector, using a wavelength of 1.0000 Å, a Δϕ of 1° and an exposure time of 35 s per image.

Data was integrated with MOSFLM 7.0.4 [31] or XDS [32] and scaled with the program SCALA [33] from the CCP4 suite (Collaborative Computational Project, 1994). Structures of complexes were solved by molecular replacement with the software MOLREP [34] using the coordinates of a whole tetramer of *Tb*PTR1 (PDB code 2X9G) and *Lm*PTR1 (PDB code 2BFA) as searching models. In all models, non-protein atoms and water molecules were omitted. Structures were refined using REFMAC5 [35]. The refinement protocol consisted of a sequence of iterative manual rebuilding of the models and maximum likelihood refinement. Visual inspection, manual rebuilding of the models and modeling of the missing atoms into the electron density between refinement cycles were performed with Coot [36]. Water molecules were added using default parameters as implemented in the software ARP/wARP suite [37] and checked by visual inspection [38].

The final models were inspected manually and checked with both Coot and Prockeck [39]. Data collections and refinement statistics are reported in Tables S2 and S3.

Figures of the electron density were generated using CCP4mg [40].

PDB codes: 5K6A (*Tb*PTR1 in complex with compound **1**), 5L4N (*Lm*PTR1 in complex with compound **1**), 5L42 (*Lm*PTR1 in complex with compound **3**).

### 3.8. In Vitro Evaluation of Activity against L. infantum Intramacrophage Amastigotes

The efficacy of compounds against *L. infantum* (MHOM/BR/1972/BH46) intracellular amastigotes was measured at a single concentration of 10 µM. THP-1 cells were plated onto 384-well plates in Roswell Park Memorial Institute (RPMI) 1640 medium, Hyclone, GE Healthcare Life Sciences (Issaquah, WA, USA) containing 50 ng/mL phorbol 12-myristate 13-acetate (PMA, Sigma-Aldrich, Saint Louis, MS, USA), and incubated for 48 h. Then, 6 day-old promastigotes were added, at a ratio of 50 parasites per each THP-1 cell seeded earlier. After 24 h of infection, negative controls (0.5% DMSO), positive controls (10 μM amphotericin B) or compounds were added to the plate. Assay plates were incubated for 48 h, and then fixed with 4% paraformaldehyde and stained with Draq 5. The Operetta high-content automated imaging system was used to acquire images and the Harmony Software was optimized to quantify the host cell number, the infection ratio and the number of parasites per infected cell. The ratio between infected cells and total number of cells is then calculated, and defined as the Infection Ratio (IR). The raw data for IR values was normalized to negative—DMSO (mock)-treated infected cells—and positive (non-infected cells) controls to determine the normalized antiparasitic activity [41].

### 3.9. In Vitro Evaluation of Activity against T. brucei

The efficacy of compounds against *T. brucei* bloodstream forms was evaluated using a modified resazurin-based assay previously described in literature [42].

### 3.10. Cytotoxicity Assessment against THP-1 Macrophages

The effect of compounds **1**–**3** on THP-1-derived macrophages was assessed by the colorimetric (MTT) assay (3-(4,5-dimethylthiazol-2-yl)-2,5-diphenyl tetrazolium bromide).

### 3.11. hERG Assay

The assay made use of Invitrogen’s Predictor™ hERG Fluorescence Polarisation Assay. This uses a membrane fraction containing hERG channel (Predictor™ hERG Membrane) and a high-affinity red fluorescent hERG channel ligand, or “tracer” (Predictor™ hERG Tracer Red), whose displacement by test compounds can be determined in a homogenous, Fluorescence Polarisation (FP)-based format [43].

### 3.12. Cytochrome P450 1A2, 2C9, 2C19, 2D6 and 3A4 Assays

These assays made use of the PromegaP450-Glo™ (Madison, AL, USA) assay platform. Each CYP450 assay includes microsomal preparations of cytochromes from baculovirus-infected insect cells. Action of the CYP450 enzymes on each substrate ultimately resulted in the generation of light and a decrease in this was indicative of inhibition of the enzymes [44].

### 3.13. Cytotoxicity Assay against A549 and W1-38 Cells

The assays were performed using the CellTiter-Glo assay from Promega (Madison, AL, USA). The assay detects cellular adenosine triphosphate (ATP) content with the amount of ATP being directly proportional to the number of cells present. The A549 cells were obtained from DSMZ (German Collection of Microorganisms and Cell Cultures, Braunschweig, Germany) and W1-38 cells were obtained from ATCC (ATCC^®^ CCL-75™) and were grown in Dulbecco's Modified Eagle Medium (DMEM) from Capricorn Scientific GmbH, Ebsdorfergrund, Germany with fetal bovine serum (FBS) (10% *v*/*v*) from Capricorn Scientific GmbH, Ebsdorfergrund, Germany, streptomycin (100 μg/mL) and penicillin G (100 U/mL) [45].

### 3.14. Aurora B Kinase Assay

This assay made use of the ADP-Glo Kinase Enzyme System from Promega (Madison, AL, USA), which is a bioluminescent assay that employs firefly luciferase in a coupled-enzyme assay format to enable detection of adenosine diphosphate (ADP) levels from ATPase assays [46].

### 3.15. Assessment of Mitochondrial Toxicity

This made use of MitoTracker^®^ Red chloromethyl-X-rosamine (CMXRos) uptake and High Content Imaging to monitor compound mediated mitochondrial toxicity in the 786-O (renal carcinoma) cell line. Cells were maintained using Roswell Park Memorial Institute-1640 (RPMI-1640) medium from Capricorn Scientific GmbH, Ebsdorfergrund, Germany containing 2 mM glutamine, FBS (10% *v*/*v*), streptomycin (100 μg/mL) and penicillin G (100 U/mL) [47].

## 5. Conclusions

PTR1 is a promising target for the development of antitrypanosomal and antileishmanial agents. The identification of novel scaffolds for the design and synthesis of PTR1 inhibitors is an ongoing challenge. Crystallographic structures are an essential tool for structure-based drug discovery programs, since they allow rapid scaffold optimization and hit-to-lead progression. Herein, we solved the crystal structure of the *Tb*PTR1-**1** complex. Moreover, these experiments provided the first X-ray crystallographic structures of *Lm*PTR1 in complex with chroman-4-one derivatives (compounds **1** and **3**). The chroman-4-one scaffold binds to *Tb*PTR1 and *Lm*PTR1 with an orientation similar to that of the biopterin substrate, suggesting that it is a promising scaffold for the development of PTR1 inhibitors. The compounds were assessed for early toxicity properties and were found to have a safe profile. The three synthesized compounds were evaluated against *T. brucei* and *L. infantum*. Compound **1** showed activity both against the targeted enzymes (*Tb*PTR1/*Lm*PTR1) and the parasites (*T. brucei* and *L. infantum*) together with a selectivity index greater than 7 and a low toxicity. Thus, compound **1** is a promising hit. The use of the flavanone scaffold guided by the crystal structures could lead to the design of more potent and selective PTR1 inhibitors.

## Figures and Tables

**Table 1:** IC_50_ values of compounds **1**–**3** and **1A**–**3A** against *Trypanosoma brucei* pteridine reductase 1 (*Tb*PTR1) and *Leishmania major* pteridine reductase 1 (*Lm*PTR1). Standard deviations agreed to ±10%. NI: No Inhibition. The percentage of inhibition at 50 μM is reported in Table S1 of the Supporting Information. The control compound was pyrimethamine, a PTR1 inhibitor (100% inhibition at 50 μM against both PTR1 enzymes). NI = No Inhibition (percentage of inhibition lower than 15%).

Compound	IC_50_ *Tb*PTR1 (μM)	IC_50_ *Lm*PTR1 (μM)
**1**	31.0	57.0
**2**	133.0	35.0
**3**	82.0	36.0
**1A**	4.3	12.5
**2A**	NI at 50 μM	NI at 50 μM
**3A**	38.0	35.0

## Supplementary Materials

Supplementary materials are available online.
