# Supplementary material for: Chroman-4-One Derivatives Targeting Pteridine Reductase 1 and Showing Anti-Parasitic Activity
Source: Molecules. 2017 Mar 8;22(3):426. doi: 10.3390/molecules22030426 (PMC6155272; doi:10.3390/molecules22030426)
Supplement: Supplementary file 1 [file molecules-22-00426-s001.pdf]

# Chroman-4-one derivatives targeting pteridine reductase 1 and showing anti-parasitic activity

**Flavio Di Pisa [1], Giacomo Landi [1], Lucia Dello Iacono [1], Cecilia Pozzi [1], Chiara Borsari [2], Stefania Ferrari [2], Matteo Santucci [2], Nuno Santarem [3], Anabela Cordeiro-da-Silva [3], Carolina B. Moraes [4], Laura M. Alcantara [4], Vanessa Fontana [4], Lucio H. Freitas-Junior [4,5], Sheraz Gul [6], Maria Kuzikov [6], Birte Behrens [6], Ina Pöhner [7], Rebecca C. Wade [7,8,9], Maria Paola Costi [2,*] and Stefano Mangani [1,10,*]**

## Table of contents:

**Characterization of compound 1-3.** S2

**Table S1.** Compounds inhibitory activity towards *Tb*PTR1 and *Lm*PTR1. S3

**Table S2**. Data collection and processing statistics. S4

**Table S3.**Structure solution and refinement. S5

**Table S4.** ADME-Tox data. S6

**Table S5.** Anti-parasitic activity of compounds **1-3** against *T. brucei* at 10 μM and *L. infantum* at 50 μM, $EC_{50}$ towards *T. brucei* and NOAEL. S7

**Characterization of compounds 1-3**

**6-hydroxy-2-(3-hydroxyphenyl)chroman-4-one (1)** was isolated as a yellow solid in a 26% yield. Mp 240 °C. $^{1}$H NMR (DMSO, 400 MHz) δ ppm: 9.51 (br s, 1H, 3′-OH), 9.42 (br s, 1H, 6-OH), 7.20 (dd, 1H, $J_{5',4'}$ = 8.2 Hz, $J_{5',6'}$ = 7.4 Hz, H-5′), 7.12 (d, 1H, $J_{5,7}$ = 2.9 Hz, H-5), 7.04 (dd, 1H, $J_{7,8}$ = 8.8 Hz, $J_{7,5}$ = 2.9 Hz, H-7), 6.95 (d, 1H, $J_{8,7}$ = 8.8 Hz, H-8), 6.91 (m, 2H, H-6′ + H-2′), 6.76 (d, 1H, $J_{4',5'}$ = 8.2 Hz, H-4′), 5.47 (dd, 1H, $J_{2,3b}$ = 12.7 Hz, $J_{2,3a}$ = 2.6 Hz, H-2), 3.10 (dd, 1H, $J_{3b,3a}$ = 16.8 Hz, $J_{3b,2}$ = 12.7 Hz, Hb-3), 2.76 (dd, 1H, $J_{3a,3b}$ = 16.8 Hz, $J_{3a,2}$ = 2.6 Hz, Ha-3). $^{13}$C NMR (DMSO, 100 MHz) δ ppm: 192.17, 157.90, 154.81, 152.03, 141.11, 129.99, 124.98, 121.33, 119.46, 117.43, 115.69, 113.79, 110.39, 79.08, 44.22. ESI-HRMS calcd for $C_{15}H_{13}O_{4}$ $[M+H]^{+}$257.0808, found 257.0808.

**6-hydroxy-2-(4-hydroxyphenyl)chroman-4-one (2)** was isolated as a yellow solid in a 31% yield. Mp 230 °C. $^{1}$H NMR ($CD_{3}OD$, 400 MHz) δ ppm: 7.33 (d, 2H, $J_{2',3'/5',6'}$ = 8.6 Hz, H-2′ + H-6′), 7.22 (d, 1H, $J_{5,7}$ = 3.0 Hz, H-5), 7.04 (dd, 1H, $J_{7,8}$ = 8.9 Hz, $J_{7,5}$ = 3.0 Hz, H-7), 6.90 (d, 1H, $J_{8,7}$ = 8.9 Hz, H-8), 6.84 (d, 2H, $J_{3',2'/5',6'}$ = 8.6 Hz, H-3′ + H-5′), 5.34 (dd, 1H, $J_{2,3b}$ = 13.3 Hz, $J_{2,3a}$ = 2.8 Hz, H-2), 3.08 (dd, 1H, $J_{3b,3a}$ = 17.0 Hz, $J_{3b,2}$ = 13.3 Hz, Hb-3), 2.74 (dd, 1H, $J_{3a,3b}$ = 17.0 Hz, $J_{3a,2}$ = 2.8 Hz, Ha-3). $^{13}$C NMR ($CD_{3}OD$, 100 MHz) δ ppm: 193.46, 157.50, 155.63, 151.51, 130.09, 127.59 (2C), 124.55, 120.77, 118.76, 114.90 (2C), 109.95, 79.45, 44.00. ESI-HRMS calcd for $C_{15}H_{13}O_{4}$ $[M+H]^{+}$257.0808, found 257.0805.

**2-(3,4-dihydroxyphenyl)-6-hydroxychroman-4-one (3)** was isolated as an orange solid in a 26% yield. Mp 220 °C. $^{1}$H NMR (DMSO, 400 MHz) δ ppm: 9.35 (br s, 1H, OH), 8.90 (br s, 2H, OH), 7.11 (d, 1H, $J_{5,7}$ = 3.0 Hz, H-5), 7.02 (dd, 1H, $J_{7,8}$ = 8.8 Hz, $J_{7,5}$ = 3.0 Hz, H-7), 6.91 (d, 1H, $J_{8,7}$ = 8.8 Hz, H-8), 6.90 (m, 1H, H-2′), 6.75 (m, 2H, H-5′ + H-6′), 5.35 (dd, 1H, $J_{2,3b}$ = 12.8 Hz, $J_{2,3a}$ = 2.6 Hz, H-2), 3.10 (dd, 1H, $J_{3b,3a}$ = 16.8 Hz, $J_{3b,2}$ = 12.8 Hz, Hb-3), 2.68 (dd, 1H, $J_{3a,3b}$ = 16.8 Hz, $J_{3a,2}$ = 2.6 Hz, Ha-3). $^{13}$C NMR (DMSO, 100 MHz) δ ppm: 192.52, 154.99, 151.90, 146.01, 145.62, 130.49, 124.93, 121.26, 119.42, 118.27, 115.77, 114.74, 110.36, 79.22, 44.13. ESI-HRMS calcd for $C_{15}H_{13}O_{5}$ $[M+H]^{+}$273.0757, found 273.0759.

**Table S1.**Inhibitory activity of compounds against*Tb*PTR1 and *Lm*PTR1.

The control compound was pyrimetamine (100% of inhibition at 50 μMagainst both enzymes).

The SD values of $IC_{50}$s agreed to ± 10%.

| **Comp.** | **% Inhibition *Lm*PTR1 (50 μM)** | **SD % Inhibition *Lm*PTR1** | **$IC_{50}$ *Lm*PTR1 (μM)** | **% Inhibition *Tb*PTR1 (50 μM)** | **STD % Inhibition *Tb*PTR1** | **$IC_{50}$ *Tb*PTR1 (μM)** |
|---|---|---|---|---|---|---|
| **1** | 50.4 | 1.6 | 57.0 | 81.3 | 0.6 | 31.0 |
| **2** | 56.0 | 3.4 | 35.0 | 17.7 | 1.6 | 133.0 |
| **3** | 69.0 | 0.3 | 36.0 | 34.6 | 1.9 | 82.0 |
| **1A** | 86.2 | 0.4 | 12.5 | 96.1 | 0.1 | 4.3 |
| **2A** | 10.8 | 1.3 | - | 2.6 | 1.5 | - |
| **3A** | 75.4 | 0.6 | 35.0 | 53.1 | 1.0 | 38.0 |

**Table S2**. Data collection and processing statistics. Values for the outer shell are given in parentheses.

| | ***Lm*PTR1+NADP⁺** | | ***Tb*PTR1+NADP⁺** |
|---|---|---|---|
| | **Compound 1** | **Compound 3** | **Compound 1** |
| PDB code | **5L4N** | **5L42** | **5K6A** |
| Diffraction source | DLS-I04 | XRD-1 | XRD-1 |
| Wavelength (Å) | 0.9795 | 1.0000 | 1.0000 |
| Temperature (K) | 100 | 100 | 100 |
| Detector | Pilatus 6M-F | Pilatus 2M | Pilatus 2M |
| Rotation range per image (°) | 0.2 | 1 | 1 |
| Exposure time per image (s) | 0.2 | 35 | 15 |
| Space group | $P2_12_12_1$ | $P2_12_12_1$ | $P2_1$ |
| No. of subunit in ASU | 4 | 4 | 4 |
| *a*, *b*, *c* (Å)<br>β (°), for monoclinic cell | 94.70, 104.25, 137.04 | 94.70, 104.19, 137.04 | 74.72, 89.89, 82.68<br>115.75 |
| Resolution range (Å) | 94.70-2.35 (2.48-2.35) | 47.35-2.10 (2.21-2.10) | 41.74-1.70 (1.79-1.70) |
| Total No. of reflections | 186708 (26441) | 328980 (47347) | 225595 (31568) |
| No. of unique reflections | 54151 (7950) | 79156 (11386) | 100585 (14665) |
| Completeness (%) | 95.3 (97.1) | 99.5 (99.5) | 93.0 (93.3) |
| Redundancy | 3.4 (3.3) | 4.2(4.2) | 2.2 (2.2) |
| $\langle I/\sigma(I)\rangle$ | 7.5 (2.7) | 5.6 (2.9) | 6.8 (1.9) |
| $R_{rmeas}$† | 15.3 (51.6) | 15.3 (33.1) | 11.2 (42.4) |
| Overall *B* factor from Wilson plot (Å²) | 17.8 | 13.6 | 7.1 |

**Table S3.**Structure solution and refinement. Values for the outer shell are given in parentheses.

| | **LmPTR1+NADP+** | | **TbPTR1+NADP+** |
|---|---|---|---|
| | **Compound 1** | **Compound 3** | **Compound 1** |
| PDB code | **5L4N** | **5L42** | **5K6A** |
| Resolution range (Å) | 82.97-2.35 (2.41-2.35) | 47.35-2.10 (2.16-2.10) | 41.74-1.70 (1.74-1.70) |
| Completeness (%) | 94.61 (96.86) | 99.22 (99.36) | 92.66 (91.37) |
| No. of reflections, working set | 51393 (3869) | 75089 (5450) | 95660 (6902) |
| No. of reflections, test set | 2714 (168) | 3968 (289) | 4859 (365) |
| Final $R_{cryst}$ | 16.6 (22.4) | 16.7 (18.3) | 18.5 (30.9) |
| Final $R_{free}$ | 21.8 (26.9) | 21.4 (21.7) | 22.7 (32.5) |
| Cruickshank DPI | 0.28 | 0.17 | 0.12 |
| No. of non-H atoms | | | |
| Protein | 7739 | 7828 | 7336 |
| Ligand | 266 | 354 | 276 |
| Water | 563 | 843 | 775 |
| Total | 8568 | 9025 | 8387 |
| R.m.s. deviations | | | |
| Bonds (Å) | 0.017 | 0.019 | 0.017 |
| Angles (°) | 1.878 | 1.955 | 1.897 |
| Average *B* factors (Å$^2$) | 24.15 | 22.84 | 19.86 |
| Estimate error on coordinates based on R value (Å ) | 0.28 | 0.17 | 0.12 |
| Ramachandran plot | | | |
| Most favored (%) | 95.0 | 95.0 | 97.0 |
| Allowed (%) | 5.0 | 5.0 | 3.0 |

**Table S4.** ADME-Tox data.

* A549 and W1-38 cell growth: 100% = not cytotoxic, 0% = cytostatic. ** Mitochondrial toxicity:100% = mitotoxic, 0% = not mitotoxic.
NI = no inhibition
- Not measured
The SD values of $IC_{50}$s agreed to ± 10%.

| Comp. | % Cell growth A549* (10 µM) | SD % Cell growth A549 | $GIC_{50}$ A549 (µM) | % Cell growth W1-38* (10 µM) | SD % Cell growth W1-38 | $GIC_{50}$ W1-38 (µM) |
|---|---|---|---|---|---|---|
| **1** | 137 | 7 | > 100 | 108 | 29 | 84 |
| **2** | 105 | 9 | > 100 | 91 | 11 | > 100 |
| **3** | 124 | 4 | 93 | 86 | 6 | 41 |

| Comp. | % Mitochondrial toxicity ** | SD % Mitochondrial toxicity (10 µM) | $IC_{50}$ Mitochondria (µM) | % Inhibition Aurora B kinase (10 µM) | SD % Inhibition Aurora B kinase |
|---|---|---|---|---|---|
| **1** | 6 | 4 | > 100 | NI | - |
| **2** | NI | - | > 100 | NI | - |
| **3** | NI | - | > 100 | NI | - |

| Comp. | % Inhibition hERG (10 µM) | SD % Inhibition hERG | $IC_{50}$ hERG (µM) | % Inhibition CYP1A2 (10 µM) | SD % Inhibition CYP1A2 | $IC_{50}$ CYP1A2 (µM) |
|---|---|---|---|---|---|---|
| **1** | 7 | 2 | > 100 | 3 | 6 | 29 |
| **2** | 14 | 8 | > 100 | NI | - | 59 |
| **3** | 20 | 8 | 64 | NI | - | 62 |

| Comp. | % Inhibition CYP2C9 (10 µM) | SD % Inhibition CYP2C9 | $IC_{50}$ CYP2C9 (µM) | % Inhibition CYP2C19 (10 µM) | SD % Inhibition CYP2C19 | $IC_{50}$ CYP2C19 (µM) |
|---|---|---|---|---|---|---|
| **1** | NI | - | 72 | 77.15 | 3.06 | 1 |
| **2** | NI | - | > 100 | 44.54 | 5.51 | 4 |
| **3** | NI | - | 4 | 2.68 | 11.85 | 5 |

| Comp. | % Inhibition CYP2D6 (10 µM) | SD % Inhibition CYP2D6 | $IC_{50}$ CYP2D6 (µM) | % Inhibition CYP3A4 (10 µM) | SD % Inhibition CYP3A4 | $IC_{50}$ CYP3A4 (µM) |
|---|---|---|---|---|---|---|
| **1** | NI | - | 85 | NI | - | 80 |
| **2** | NI | - | 94 | NI | - | > 100 |
| **3** | NI | - | > 100 | NI | - | 65 |

**Table S5.** Anti-parasitic activity of compounds **1-3** against *T. brucei* at 10 μM and *L. infantum* at 50 μM, $EC_{50}$ towards *T. brucei* and NOAEL. The reference compounds were pentamidine ($IC_{50}$ = 1.55 ± 0.24 nM) for *T. brucei* and miltefosine ($IC_{50}$ = 2.65 ± 0.40 μM) for *L. infantum*. The SD values of $IC_{50}$s agreed to ± 10%. ND: Not Determined

| **Comp.** | % inh. *L. infantum* (50 μM) | % inh. *T. brucei* (10 μM) | $EC_{50}$ *T. brucei* | $CC_{50}$ ± SD or NOAEL |
|---|---|---|---|---|
| **1** | 31 | 44 | 12.58 ± 1.69 | >100 |
| **2** | 29 | 49 | 13.02 ± 1.82 | >100 |
| **3** | 3 | 14 | 34.82 ± 1.10 | >100 |
